# Re‐opening Pandora's box: Who owns professionalism and is it time for a 21st century definition?

**DOI:** 10.1111/medu.14862

**Published:** 2022-07-03

**Authors:** Viktoria C. T. Goddard, Susannah Brockbank

**Affiliations:** ^1^ School of Medicine University of Liverpool Liverpool UK

## Abstract

**Background:**

The concept of professionalism is dominant within health care education and the lives of practising clinicians globally, and yet there is no single agreed definition nor framework applied universally across the health care professions. This article questions how much attention is paid to where definitions of the concept of professionalism came from and whether the accepted norms within the dominant discourses are still truly applicable to a 21st century workforce.

**Method:**

Taking a critical look at the existing body of literature on professionalism using a locus of medical education, this article reviews who the dominant voices have been in the creation of current understandings of professionalism. Using a pragmatic and targeted approach, regulatory body definitions of professionalism from across the world are compared to demonstrate the complexities of finding a universally accepted definition of the concept.

**Results:**

The article suggests that the extant definitions are grounded but also stuck in a background of Western, White, heteronormative view of society and the professions of the past and argues that we need to better understand the expectations of professionalism from the perspectives of those who now work in health care and health care education, as well as reviewing the views of the “public” within this debate.

**Conclusions:**

By reopening the “Pandora's box” of professionalism, this article argues that we can improve the quality of definitions—and thus application—of professionalism for health care professionals and patients.

## Abstract 2

Goddard and Brockbank question where extant definitions of professionalism come from while querying it it is time to move away from the existing canon.

## 1 INTRODUCTION

“[P]*rofessionalism is a successful ideology* and as such has entered the political vocabulary of a wide range of occupational groups who compete for status and income”.^1^ (emphasis added)

Graduating as a health‐care practitioner involves the development of a new professional identity,^1^ a concept that matters precisely because of the status and privilege that is acquired with gaining such an identity.^2^ The challenge that befalls the (often) young people graduating into the health‐care professions is that with this identity comes a set of expectations, observed by their educational establishments, on behalf of the relevant regulatory bodies, enforced on behalf of society: professionalism. “Professionalism” then is a dominant concept in health‐care education and in the life of any health‐care student.

We have been researching and publishing in the area of medical professionalism and identity development for some years,^1^, ^3^, ^4^, ^5^ but it still feels as if we are relative newcomers to the field. Our shared reflection from discussing challenges concerning professionalism with global colleagues^6^, ^7^, ^8^ is that we are not alone in this; in order to understand and define professionalism, we all draw upon the same set of authors as recognised canon across the health professions.^9^, ^10^ This canon itself is usually based in medicine; notably, as one of the first defined professions alongside law and the priesthood, there has arguably been longer to debate the concept of professionalism in this field than for the more newly “professionalised” health‐care occupations, although there is no shortage of work related to positioning professionalism in other health‐care professions.^11^, ^12^

Yet despite a shared frame of reference for understanding “professionalism”, when we discuss scenarios of professionalism transgressions, there is never one agreed approach to remediation nor prevention of further transgressions.^4^ We often talk about the importance of social or cultural context; and we reflect that the published literature on professionalism does not seem to represent our own thoughts and values. Dominant definitions seem to ignore considerations of gender, ethnicity, twenty‐first century expectations of work‐life balance and often, most worryingly of all, physical and mental health concerns.

Meanwhile outside health care, these considerations appear to already exist in discussions of professionalism.^13^, ^14^ Indeed, our concerns mirror Friedson's own reflections^15^ in defining a “profession” 40 years ago. Friedson noted the folly of attempting to define the term as a “generic concept” rather than acknowledging it is a “changing historic concept with particularistic roots in those in industrial nations that are strongly influenced by Anglo‐American institutions” (p32). With this in mind, we feel it is beyond time to review the definitions of professionalism to understand how they are applied in different contexts. Our own work here will once again be based in medicine; thus whilst we speak of these debates as relevant to all health‐care professions, we must acknowledge the context of each singular health‐care profession may mean this debate needs to be had specifically for each occupational identity.

It is also important to situate this discussion within this special issue on the theme of quality improvement. As educators, we find ourselves constantly striving to improve the quality of our professionalism teaching, as well as searching for optimal methods to manage professionalism lapses amongst our students.^4^ We often, however, find ourselves hamstrung by definitions of professionalism that are simply not up to the task. Thus, in our eyes, the first step to improving quality in teaching and management of professionalism is to encourage an open debate of existing definitions and to interrogate the origins and assumptions that underpin these.

In order to undertake a critical appraisal of the current state of medical professionalism, we will seek to address the following questions:

- who are the dominant voices that are “allowed” to define professionalism? Should defining professionalism be the remit of regulators, academics, the profession or indeed the amorphous “public”?
- If the dominant voices do not speak for all – or even a majority – within a profession, what does that mean for hegemonic definitions of professionalism and for the subsequent delineation of professional standards?
- Taking into account different understandings across different national and cultural contexts, is it even possible for there to be one single accepted definition of professionalism that “works” for everyone?
- Is the danger that uncritical acceptance of current definitions of professionalism results in the concept being weaponised and used against the professions themselves?

This paper explores the debate on each of these questions, to make the case that these conversations need to move on from the position that the extant definitions of professionalism are the definitive ones.

## 2 WHO IS – AND SHOULD BE – ALLOWED TO DEFINE PROFESSIONALISM?

Attempts to define a “profession” and thus professionalism started in the field of sociology; most broadly, professionalism is the aspects of occupations that justify members' position in society.^16^ From these definitions emerged the concept of a “social contract”,^17^ that is where professionals have access to knowledge and skills that are useful to society. This knowledge is safeguarded by the profession itself – so is not accessible to all – thus, in order to be trusted to wield said knowledge and continue to regulate access to it, doctors must behave in a way that justifies the trust of those outside the profession. Immediately this suggests that professionalism is defined by members of the public: only those out with the profession can define what would justify their trust. This is, however, clearly not the case: even this article, existing within the field of medical education, demonstrates that the debate regarding professionalism is one dominated by doctors. Thus, the question arises: which members of the profession claim the power to define professionalism?

### 2.1 Academics

#### 2.1.1 Professionalism is the basis of medicine's contract with society^18^

As this quotation suggests, much of the academic literature that discusses professionalism cites the “social contract” as the reason why doctors must be professional.^19^, ^20^ As we have established that little is known about definitions set by the public, it must follow that the expectations of the public are only inferred from a combination of what doctors *think* members of the public expect and the public reaction to stories of malpractice that hit the headlines.^21^, ^22^ Though the “social contract” may be a useful theoretical construct, the reality is that it seems at best to be based on historical guesswork and at worst to be a fiction.

At this stage, it is perhaps important to outline some difficulties with the power of the establishment that seems to have control over the definition of professionalism and the social contract. Whilst the concept of a “profession” has been discussed since at least the 1930s,^23^ professionalism as a concept referring to the standards and practices associated with a profession emerged from British and American sociological analysis concerning the value of occupations, gaining traction towards the end of the 1970s and early 1980s.^24^, ^25^ Indeed in 1990, Burrage and Torstendahl^24^ insightfully argued that caution was needed in this field; the separation of a defined profession from its social and political context, and the neglect of inter‐professional relationships and an Anglo‐American bias were all identified as concerns in the application of the theorising of professional behaviours.

In this already heavily debated body of work, it is perhaps understandable that it feels challenging to break into scholarly discussion of professionalism. Nevertheless, this may be less to do with the time dedicated to understanding the evolution of medical professionalism and relate more to the apparent immovable dominance of particular figures in the field, and a subsequent lack of critical engagement with the concerns highlighted above. Cruess and Cruess, for example, have been publishing about definitions and teaching of professionalism for over 20 years,^9^, ^26^ and they are often cited by other authors as a kind of lodestone for defining professionalism.^27^ It is worth noting, however, that the Cruesses are a white, Canadian couple who graduated in the 1950s.^28^ With this in mind, their definitions of professionalism require some interrogation. When these definitions of professionalism were first accepted,^9^, ^18^ medicine looked very different. It was a profession still dominated – certainly in senior positions – by a very homogeneous workforce. Whilst these definitions may have been thoughtful and well theorised for their context, we believe it is increasingly challenging to apply them unquestioningly to the current and future workforce.

One of the challenges in presenting an alternative definition, however, seems to have been gaining traction in the debate. In 2006, for example, Wear and Aultman^29^ edited a collection of critical analyses of professionalism using different theoretical frames. One of these highlights the potential for gender inequality when using conventional definitions of professionalism.^30^ For example, if professionalism requires supererogation – going above and beyond the call of duty – can parents who need to leave on time to care for their children still be professional? Perhaps, unqualified commitment to medicine can only be the privilege of those supported in their personal lives by a partner who assumes responsibility for all domestic tasks. As medicine embraces an increasingly diverse workforce, surely it has become possible to leave work on time *and* be professional? And yet Shirley and Padgett's^30^ critique has been largely lost in the clamour to cite and recite the dominant definitions. This suggests to us that there is a widening gulf between the academic discussion of professionalism and what is happening “on the ground”. If this is the case, perhaps a practical definition of professionalism is more widely applicable to practising physicians.

A further challenge arises when attempting to translate the “social contract” into a commonly agreed definition.^31^ Definitions of professionalism that rely solely on behaviour have been criticised for being “skin deep”,^32^ whereas definitions that rely on virtues are amorphous and difficult to implement in assessment.^33^ Some have suggested that normative definitions – based on how *most* doctors would behave – are helpful.^34^ Pertinently, however, norms change with generations and herein lies the difficulty: the definition of professionalism is not equally controlled by all members of the profession. Instead, powerful stakeholders such as academics and regulators seem to control the definition.

### 2.2 Regulators

Perhaps the most straightforward definition of professionalism is one used by regulatory bodies. These definitions – and thus the institutions that control them – have power as they form de facto standards against which professionalism is measured. In the United Kingdom, for example, the General Medical Council (GMC) uses this definition to outline what they believe professionalism means:

“Good doctors make the care of their patients their first concern: they are competent, keep their knowledge and skills up to date, establish and maintain good relationships with patients and colleagues, are honest and trustworthy, and act with integrity and within the law.”^35^

Regulatory bodies have a powerful role in defining professionalism as they set the conditions of entry to – and legitimate employment within – medicine. Yet there are manifest problems with a definition such as this. Firstly, each term – take “good” as an example – requires further clarification. Next, each concept needs to be contextualised with a given doctors' experience, stage of training and the specific scenario at hand, all of which are challenging to measure in an objective way. This lack of clarity then opens the definition to interpretation and leaves these interpretations to be made through a kind of informal regulatory case law. The GMC's own definition of professionalism is tested through tribunals of medical practitioners, which are conducted by doctors and lay people experienced in dealing with matters of professional misconduct and fitness to practise. Thus, the “establishment” maintains its own definitions with little opportunity for external critique.

Another challenge of relying on a regulatory definition of professionalism is that it is only tested in relief: only behaviours and attitudes that *do not* meet professional standards are publicly measured against it. This is unhelpful as the “black and white” of professionalism changes little over time. It is never going to be professional to kill your patients for personal financial gain. What do change are the “shades of grey”, the decisions that are less clear, which are less often the remit of the regulator. Instead, they are decisions that fall to educators, health‐care providers, “jobbing” doctors and members of the public to make on a day‐by‐day basis.

## 3 TAKING INTO ACCOUNT DIFFERENT UNDERSTANDINGS ACROSS DIFFERENT NATIONAL AND CULTURAL CONTEXTS, IS IT EVEN POSSIBLE FOR THERE TO BE ONE SINGLE ACCEPTED DEFINITION OF PROFESSIONALISM THAT “WORKS” FOR EVERYONE?

As has been acknowledged in multiple previous publications, there is no single or agreed definition of professionalism across national or international borders.^36^ As such, each national/regional regulator or medical licensing organisation has its own regulations and to a certain extent their own definition of “professionalism”. These definitions are sometimes encapsulated in guidance incorporated into professional regulatory documents,^37^ whereas other definitions are captured in whole documents focussed on professional expectations either in undergraduate training^38^ or for registered professionals.^39^ Indeed, in some contexts definitions of professionalism seem to be generated by doctors' associations, rather than the regulatory body.^39^, ^40^ There is not then an easily identifiable set of comparable documents by which we can consider how professionalism is addressed in different national guidance, though there have been a number of attempts to do so. Most notably, a recent set of literature notes that Western definitions of professionalism do not necessarily reflect the cultural values of non‐Western countries, and therefore, that different professionalism definitions could be needed in different contexts.^41^, ^42^, ^43^, ^44^, ^45^, ^46^, ^47^, ^48^, ^49^, ^50^

Table 1 attempts to bring together a small representation of guidance that emerges if one searches for “professionalism” on the regulatory or accreditation body websites. Within the confines of an article of this length, it was not possible to search for and include an example from every country where medicine is practised as a regulated profession. Instead we chose to include a cross‐section as far as we could across continents to demonstrate the complexities of defining professionalism. The search for this information was therefore deliberately pragmatic and included locations from where we knew large bodies of medical education literature already had representation on the international stage (UK, Europe, USA and Australia – what might previously have been termed the “Western” voice) and then others less well represented (China, Saudi Arabia, South Africa and Japan). However, we acknowledge this further amplifies the already dominant voices in the field and leaves the underrepresented nations still unheard. This highlights one of the reasons these debates are needed: to acknowledge there are whole nations whose views on “professionalism” are unknown to those who use it as if it were a clearly defined and universally understood term.

**TABLE 1:** A cross‐national comparison of available definitions of, or guidance related to, professionalism

Country/region	Source	Excerpts of requirements/guidance on professionalism/professional behaviours
Australia	Australian Medical Council (AMC)^36^	Nothing articulated in training standards. AMC promotes a published guide on “Good Medical Practice: Professionalism, Ethics and the Law”: Professionalism covers a wide range of elements, including good communication skills, an empathetic attitude, the virtues of self‐reflection, truthfulness and dependability, cultural awareness in our multicultural society, and awareness of relevant laws pertaining to medical practice. Above all it covers an assumption that a person wishing to practise medicine effectively will bring positive attitudes to all the roles involved in being a doctor
China	National Health Commission of the Peoples Republic of China website^51^; Chinese Medical Doctor Association (CMDA) charter^52^	Nothing returned in search for “professionalism” on National Health Commission (main national health agency) website. New legislation to “better protect doctors” introduced in August 2021 clarifies that doctors must not produce false medical certification documents and not perform unnecessary examinations and treatments on patients. CMDA defines “six tenets” of medical practice: equality and benevolence; primacy of patients; honesty and fidelity to promises; commitment to excellence and prudence; incorruptibility and impartiality; lifelong learning.
Germany	German Medical Association^53^	Publishes a (Model) Professional Code for Physicians in Germany which contains the “Rules for Professional Practice”. Excerpts:
		Physicians serve the health of the individual and of the population. The medical profession is not a trade. It is by nature a liberal profession.
		Physicians practice their profession according to their conscience, the precepts of medical ethics and humaneness. They may not acknowledge any principles, or comply with any regulations or instructions, that are irreconcilable with their tasks or for whose observance they cannot answer.
		If physicians who are permanently established or perform their professional activity in another Member State of the European Union temporarily and occasionally perform their medical activity on a cross‐border basis in the territory covered by this Professional Code without establishing a practice, they must observe the provisions of this Professional Code.
		In addition to practising their profession, physicians are forbidden to engage in any other activity that is irreconcilable with the ethical principles of the medical profession. Physicians are also forbidden to allow their name to be used in conjunction with a medical occupational title in an unfair manner for commercial purposes. They may equally not permit use to be made of their name, or of the professional reputation of physicians, in such a way.
Japan	Japan Medical Association Website^39^	“… the physician should serve society with a basic love for humanity.” Underpinned by the following six principles: continuing education; dignity and responsibility, striving “to enhance his/her cultural refinement, education, and integrity”; respecting the individuality of patients, treating them with compassion and earning their trust; maintaining respect for his/her fellow physician and serving “the cause of medical care to the best of his/her abilities”; respecting the spirit of public service and contributing to the development of society; not engaging in profit‐making medical activities.
Saudi Arabia	Kingdom of Saudi Arabia Education & Training Evaluation Commission (ETEC) website^54^	SaudiMeds Framework developed by Saudi Medical Deans Committee; Theme V – Professionalism: “The commitment to deliver the highest standards of ethical and professional behaviour in all aspects of health practice, and take a responsibility for own personal and professional development. Adhere to professional attitudes and behaviours of physicians Apply Islamic, legal and ethical principles in professional practice Demonstrate the capacity for self‐reflection and professional development”
South America	Centeno et al 2016^55^	Nothing obvious available in English but Centeno et al. 2016 discuss a collaboration between three South American Schools with a shared aim to promote professionalism amongst their students; the agreed definition of professionalism used was Epstein and Hunderts^56^ definition where professionalism is: “The habitual and judicious use of communication, knowledge, technical skills, clinical reasoning, emotions, values, and reflection in daily practice for the benefit of the individual and the community being served”
South Africa	Health Professions Council of South Africa website^57^	Health Professions Council publishes a lengthy document with very specific requirements of *all* registered health‐care practitioners. An example of this is that they provide guidance on a practitioners stationery as follows:
		“4. (1) A practitioner shall print or have printed on letterheads, account forms and electronic stationery information pertaining only to such practitioners – (a) name; (b) profession; (c) registered category; (d) speciality or subspeciality or field of professional practice (if any); (e) registered qualifications or other academic qualifications or honorary degrees in abbreviated form; (f) registration number; (g) addresses (including email address); (h) telephone and fax numbers; (i) practice or consultation hours; (j) practice code number; and (k) dispensing licence number (if any).”
The Netherlands	Royal Dutch Medical Association (KNMG)^40^	KNMG Manifesto on Medical Professionalism “The aim of this manifesto is to perpetuate and promote the trust that society should be able to place in medical professionals.
		… A good doctor is a medical professional: he is a medical expert, he keeps his professional know‐how up‐to‐date, communicates in an empathic way with his patients, cooperates with colleagues, exercises his profession within the (moral) boundaries of the professional group, organises quality in his practice and renders account.
		The doctor needs society to place trust in him. Trust is necessary for the proper operation of the health‐care sector as a whole, for the doctor‐patient relationship and for the intrinsic motivation of medical professionals. The medical professionals intrinsic motivation is the most powerful driving force for qualitatively sound, meaningful and efficient care.”
United Kingdom	General Medical Council Website	*Outcomes for Graduates* ^38^ defines the standards which all medical graduates are expected to reach by the end of their pre‐registration training. It states that “newly qualified doctors must behave according to ethical and professional responsibilities” and gives a 28‐point list detailing specific requirements including: demonstrate compassionate professional behaviour and their professional responsibilities in making sure the fundamental needs of patients are addressed act with integrity, be polite, considerate, trustworthy and honest recognise the potential impact of their attitudes, values, beliefs, perceptions and personal biases (which may be unconscious) on individuals and groups and identify personal strategies to address this
		*Good Medical Practice*,^35^ a document defining professional values and behaviours expected of any qualified doctor registered with the GMC, states that: “As a good doctor you will: make the care of your patient your first concern be competent and keep your professional knowledge and skills up to date take prompt action if you think patient safety is being compromised establish and maintain good partnerships with your patients and colleagues maintain trust in you and the profession by being open, honest and acting with integrity”
United States of America	Accreditation Council for Graduate Medical Education Website^58^	Common Programme Requirements for residency training includes a long list related to professionalism, including but not limited to: “Residents and faculty members must demonstrate an understanding of their personal role in the: provision of patient‐ and family‐centred care; safety and welfare of patients entrusted to their care, including the ability to report unsafe conditions and adverse events; assurance of their fitness for work, including: management of their time before, during, and after clinical assignments; and, recognition of impairment, including from illness, fatigue, and substance use, in themselves, their peers, and other members of the health‐care team. commitment to lifelong learning; monitoring of their patient care performance improvement indicators; and, accurate reporting of clinical and educational work hours, patient outcomes, and clinical experience data …
		“All residents and faculty members must demonstrate responsiveness to patient needs that supersedes self‐interest. This includes the recognition that under certain circumstances, the best interests of the patient may be served by transitioning that patients care to another qualified and rested provider …
		“Programmes, in partnership with their Sponsoring Institutions, should have a process for education of residents and faculty regarding unprofessional behaviour and a confidential process for reporting, investigating, and addressing such concerns.”

In some instances, these are translated versions, and we acknowledge a limitation here is that we were only able to access resources that were available in English, sometimes via secondary sources. We therefore do not claim this as a definitive representation of all international guidance available on professionalism, and we acknowledge there are some large gaps; the inwardly facing medical education system in Russia for example locates all key literature in Russia; therefore, we can make no claims to knowledge about attitudes to medical professionalism in this part of the world, nor anywhere else not included in the table. What it does, however, is give a sample of the types of expectations and requirements made available on national agency websites for the purpose of demonstrating how complex it is for anyone to make sense of what “professionalism” means in different contexts.

It is understandable that each national regulator will have its own standards, an important part of ensuring medicine and health care is relevant to its own context.^59^ What Table 1 demonstrates is quite how far apart the definition, application and regulation of “professionalism” is. Despite these differences, we speak and write about it within medical education as if it is just one singular concept. Interestingly, in most contexts, guidance incorporates the import of lifelong learning and/or professional development but here the direct comparison ends. The guidance from The Netherlands from the Royal Dutch Medical Association,^40^ a professional organisation rather than a regulator, follows an academic discourse and focusses on the relationship between professional status and the trust that society puts in the profession. For some regulators^38^, ^58^ there is more evidence of a continuum of guidance from pre‐registration to postgraduate training including guidance for training providers. For others,^57^ there is an incredibly detailed list of instructions leaving it hard to imagine room for application of professional judgement to individual scenarios. Notably, it is extremely challenging to imagine how the tenets of one country would apply in another; the guidance from Japan that medical activities will not be undertaken for “profit‐making” would not work in many countries where medical insurance firms exist for that very purpose, whilst reference to adherence to Islamic practice in the Saudi Arabian context^54^ makes little sense for non‐Islamic countries. Indeed, in many countries where religion is deliberately separated from the state and therefore health service provision, the inclusion of a mention of a specific religion as part of professional practices is quite jarring. Where the expectation in one country that health care activity is paused for Friday prayers is seen as professional; in another cultural context, it might be seen as unprofessional to consider a “break” from patient care for religious observance. Whilst individual national guidance may originate in superficially “similar” understandings of professionalism, the resultant application of these is much further apart, which has implications for the movement of students and staff across international boundaries. This suggests that dominant definitions are not universally applicable in the context of globalised medical practice, creating uncertainty for doctors when attempting to understand professional standards. Indeed, the German guidance specifies that, where their doctors are temporarily working on a cross‐border basis, their physicians must still follow the German rules, which raises questions for whose jurisdiction any accusations of “unprofessional” behaviours would be arbitrated by.^53^

What seems clear from our analysis is that, even within a relatively small sample of professional regulators, a universal definition of professionalism that transcends borders, cultures and contexts does not exist. Indeed, we would argue that this is not desirable as in all likelihood any attempt at a universal definition would be dominated by a Western‐centric view of the world and thus would not be representative of the needs of many doctors and indeed their patients.

## 4 IF THE DOMINANT VOICES DO NOT SPEAK FOR ALL – OR EVEN A MAJORITY – WITHIN A PROFESSION, WHAT DOES THAT MEAN FOR HEGEMONIC DEFINITIONS OF PROFESSIONALISM AND FOR THE SUBSEQUENT DELINEATION OF PROFESSIONAL STANDARDS?

One of the forces for change in the professionalism debate over recent decades has been the rise of social media. Where many doctors may have been excluded from academic and regulatory conversations, platforms such as Twitter have allowed individuals to become involved in the discussion, often in a very public way. Developing new professional relationships outside of local working environments was previously limited to conference networking, but social media platforms have changed the nature of what it means to have a public profile and professional identity as a doctor.^60^ Social media debates have often included discussions of controversial views and publications. For example, in 2020, the Journal of Vascular Surgery published a paper entitled “prevalence of unprofessional social media content amongst young vascular surgeons“. In this article, the authors implied that posting a picture of oneself on social media wearing a bikini was unprofessional.^61^ The backlash on social media from doctors around the world – employing the hashtag “MedBikini” – was so impactful; the journal was forced to retract the paper shortly thereafter. In many ways, social media platforms have allowed a new generation of doctors to claim ownership of their own definition of professionalism, not just in their online presence but also in their ability to have relatively uncensored discussion of what professionalism means to them.

Interestingly, the use of social media is itself a contentious area in relation to professionalism where what is viewed as ethical can be down to individual interpretation.^62^ Despite the fact that the American Medical Association introduced social media guidelines as long ago as 2010,^63^ and the existence of tips papers on using social media as a medical educator,^64^ regulators have not seemed to keep pace with the rapidly evolving world of social media and “e‐professionalism”.^56^, ^65^ It is increasingly unclear how “professional” it is possible to be whilst engaging online at all and there seems to be little consensus on what “professional” behaviours online looks like.^66^ Take the debate around vaccines as an example: is it unconventional, or unprofessional, for a health‐care worker to discourage individuals from receiving vaccines? Furthermore, if unconventional beliefs are construed as unprofessional, it is important to understand who is responsible for policing these views. Medical regulators do have a social media presence, but these currently exist for promotion rather than regulatory purposes. In this case, perhaps social media offers medical professionals the opportunity to be “tried by a jury of their peers”. In academic terms, this aligns with a normative definition of (e‐)professionalism, with the most commonly held ideas controlling what is deemed professional. The difficulty here, however, is that unconventional ideas may be disruptive – but might not be unprofessional. In addition, there is a line that is difficult to draw between professionalism and freedom of speech. Is it unprofessional, for example, to voice far right political opinions? Whilst we personally would struggle to see how these views are compatible with the professional values of doctors' organisations/regulators, it is unclear whether expressing an opinion in this vein amounts to unprofessional behaviour.

Increasingly, social media is used to share examples of where the concept of “professionalism” has been (mis‐)used to police appearance, language and other personal attributes. Worryingly, not only do examples include wearing of trainers (which make sense for people working on their feet all day), not wearing a tie (which is banned under infection control measures in the UK), wearing make‐up, dyed hair and, in one example, a criticism of an “unprofessional” name.^67^ This has understandably led to increasing concern from within the community and indeed from those training to join the profession that the notion of professionalism is being “weaponised”, with fellow “professionals” using social media to accuse one another of unprofessional behaviour based on differing world views and opinions.^68^ Examples have arguably always existed; however, social media offers the profession new ways of sharing, discussing and amplifying these debates.

A recent prominent example of the weaponisation of the term professionalism emerged from a medical student at the University of Newcastle (UK). This student received a penalty in a practical examination based on the length of her skirt. Feeling aggrieved, she took to Twitter armed with the story and a picture of the said skirt, attracting widespread criticism of the individual who issued the penalty and the medical school that upheld it.^69^ This demonstrates that professionalism can no longer be unquestioningly policed by the clinical and academic elite: students and trainees have a voice that cannot, and should not, be silenced.

Nevertheless, one of the interesting aspects of taking the professionalism debate to social media is that it is in the public domain. If we return to the idea of the “social contract”, perhaps social media offer a new opportunity to include the public in the debate about what professionalism means to them. Ultimately, however important the academic and regulatory debates may seem, they largely exclude the voices of “lay” people, closing the debate down and ensuring it is insular and out‐of‐step with society at large.

## 5 CONCLUSIONS

In this article, we have posed more questions than we have provided answers. Our intention was to challenge dominant discourses around professionalism and demonstrate that the concept is no longer as straightforward as it once appeared. Through this article, we hope to reinvigorate conversations about the definition of professionalism that engage widely with stakeholders in order to improve the quality of the academic conversation. We want to make a bold statement: that seeking a single global definition is pointless. As we have discussed, cultural context is so important to our understanding of professionalism. This means that a high‐quality definition of professionalism around the world is one that is culturally compatible and emotionally intelligent to the needs of each society.

In the interests of brevity, there are several issues that we have failed to include in our discussion. One example is the potential socioeconomic inequity built into the construct of professionalism: there is perhaps an extent to which unconscious awareness of particular social rules or cues makes access to professionalism easier for some individuals than others.^4^ Furthermore, when professionalism problems arise, wealth matters as it provides access to expensive defence lawyers and thus a degree of protection from adverse consequences. These are, however, bigger ideas than we can give space to here, so we invite the debate to continue.

Finally, if we are going to continue to base understandings of professionalism on the “social contract”, a marker of quality needs to be how the “public” are consulted about their expectations of medical professionals. There needs to be an ongoing two‐way discourse between the “profession” and the “public” so that professionalism can evolve in line with changing societies. We hope that this may help us wrest control of professionalism from an academic – and often Western, white, hetero‐normative, male – elite and allow the definition to pivot towards one that is more representative of the profession and society more widely. So, in our context, if professionalism is to be “weaponised”, we hope that this will be to challenge dominant ideas rather than to perpetuate them.

## FUNDING

Not applicable.

## CONFLICTS OF INTEREST

None.

## ETHICS STATEMENT

No ethical approval was required or sought for this desk‐based research article.

## AUTHOR CONTRIBUTIONS

Dr Viktoria Goddard and Dr Susannah Brockbank co‐authored this article with equal contribution to all article components.
